# Metastatic Carcinoma of the Breast Presenting as Gingival Swelling in the Maxilla: A Case Report

**DOI:** 10.1155/2022/2667415

**Published:** 2022-09-19

**Authors:** Arun Sadasivan, K. Rakul Nambiar, Deepu George Mathew, Elizabeth Koshi, Roshni Ramesh, Ashna Mariya Benny

**Affiliations:** ^1^Department of Periodontics, Sree Mookambika Institute of Dental Sciences, Kulashekaram, Tamil Nadu, India; ^2^Sree Mookambika Cancer Centre, Kulasekharam, Tamil Nadu 629161, India; ^3^Department of Oral and Maxillofacial Pathology, Annoor Dental College, Muvatupuzha, Kerala, India; ^4^Department of Periodontics, Government Dental College, Trivandrum, Kerala, India

## Abstract

**Background:**

Metastatic cancers in the oral cavity are usually very rare and are usually an indication of widespread malignancy. In some cases, oral metastasis was found to be the first presentation of distant site tumours. Even though oral metastatic lesions may be found anywhere in the oral cavity, they commonly present in the posterior areas of the jaw bones. Among the soft tissues, the gingiva is the most common site. The presence of inflammation in the gingiva and the role of periodontal microbiota are suggested to play a role in the attraction of metastatic cells. The purpose of this case report is to present a rare case of metastatic breast carcinoma presenting as a gingival enlargement in the maxillary anterior region. *Case Presentation*. A 37-year-old female patient who underwent modified radical mastectomy for invasive ductal breast carcinoma reported to the dental clinic with a gingival enlargement in the anterior maxillary region. Clinical and radiographic examination showed a rapidly enlarging gingival lesion with destruction of the underlying bone. A wide excision of the entire lesion was done. Histopathological and immunohistochemical (IHC) evaluations were suggestive of infiltrating poorly differentiated adenocarcinoma.

**Conclusion:**

This case report presents a metastatic oral lesion in the maxillary anterior region of the primary breast cancer site. The young age of patient and an uncommon site of metastatic lesion are the striking features of this case. We would like to highlight the importance of a thorough clinical, radiological, and histological evaluation of any gingival swelling as it could be a metastatic lesion. IHC staining helps in the diagnosis of the primary site of metastatic carcinomas. An early diagnosis and intervention could reduce the morbidity of the lesion and improve the survival rate.

## 1. Introduction

Metastatic cancers in the oral cavity are a rare occurrence and represent less than 1% of all malignancies reported in the oral cavity [1]. The most common metastatic malignancies in women are from primary tumours in the thyroid glands, breast, colorectal region, kidneys, and genital organs [2]. Breast cancer is one of the most common malignancies affecting females. It does have a wide metastatic pattern, the most commonly reported includes those in the lungs, lymph nodes, bone, pleura, and liver [3].

Oral metastatic lesions have been more frequently reported in the mandible rather than the maxilla, with a site predilection for the posterior areas of the mandible. The presence of hematopoietic bone marrow, multiple blood vessel branching, and slow flow of blood have been speculated as reasons for the site predilection [4]. Clinically, oral metastases often present as areas of inflammation, pain, paraesthesia, or swelling. They may often be misdiagnosed as giant cell granulomas, pyogenic granulomas, bone cysts, osteomyelitis, Paget's disease, and fibrous epulis among the differential diagnoses [5]. Only about 33% of oral metastatic lesions are found in the soft tissues, with the lung being reported as the most common source [4]. In soft tissues, the attached gingiva, alveolar mucosa, and the tongue are said to be the most common sites for metastasis [6]. In a study by Allon et al. in 2014 demonstrated a significant association between the presence of teeth and gingival metastatic lesions. In the presence of teeth, 80% of the patients had gingival metastases compared to one-third of the edentulous patients [7]. Hirshberg et al. in 1993 have suggested an important role for inflammation in attracting metastatic tumour cells to the gingiva [4]. Gingival metastatic lesions typically present as highly vascular exophytic lesions, which strongly resemble reactive or hyperplastic lesions such as fibroma, pyogenic granulomas, peripheral giant cell granulomas or fibrous epulis [7, 8]. Akheel et al. in 2013 have reported that in about 25% of cases, oral lesions are found to be the first sign of metastatic spread and in 23% of cases; oral metastatic lesions could be an indication of an unidentified primary tumour at a distant site [9].

Most often, metastases to the oral cavity occur in the end stages of cancer lesions and cause severe morbidity to the patients. This is often considered as a sign of widespread cancer and is an indicator of poor prognosis, with a 5-year survival rate below 5% [6]. Due to these reasons, it is important to detect oral metastases early. They need to be considered in the differential diagnosis of inflammatory and reactive lesions of the gingiva [4]. In this case report, we describe a case of gingival metastasis from invasive ductal carcinoma of the breast, which occurred 9 months after detection of breast cancer.

## 2. Case Presentation

A 37-year-old female patient reported to the Department of Periodontics, Sree Mookambika Institute of Dental Sciences, Kulashekaram, Tamil Nadu, India, with a chief complaint of swelling of gums in the upper front tooth region. The medical history revealed that the patient reported to the hospital 9 months back as she felt a lump on her left breast. An incision biopsy was done and was pathologically diagnosed as invasive ductal carcinoma of histological grade II. Modified radical mastectomy (lumpectomy with axillary clearance) was done to remove the lump and involved axillary lymph nodes (levels I and II). Following the surgical removal, the patient was advised to start a course of radiotherapy and chemotherapy. The patient delayed the treatment by one month and later reported to the hospital with a complaint of memory loss, following which a positron emission tomography (PET) scan was done which showed the presence of brain metastasis. Palliative whole brain radiotherapy of 3000 cGy was administered, divided into 10 fractions. While on radiation treatment, the patient presented with multiple flaccid blisters, erythematous erosions spread throughout the body and was diagnosed as Steven Johnson syndrome (probably anti-epileptic drug induced) and this was managed conservatively. The patient was then started on chemotherapy 14 days later with paclitaxel. During chemotherapy, the patient developed a swelling of the gums which was symptomatic and hence was referred to the Periodontics department for further management. The swelling occurred 9 months after the initial breast swelling was noticed.

**Figure 1:**
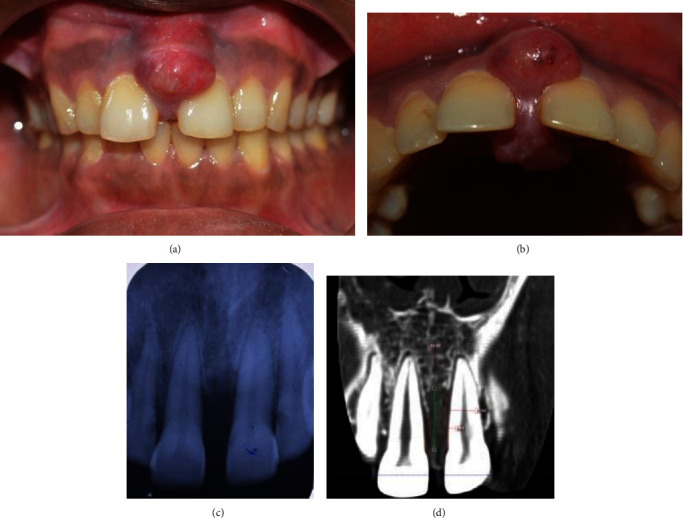
(a) and (b) Dumbbell-shaped swelling involving the buccal and palatal aspect. (c) Intra-oral peri-apical radiograph showing interdental bone loss between maxillary central incisors; (d) CBCT showing bone loss in the mid-alveolus region.

**Figure 2:**
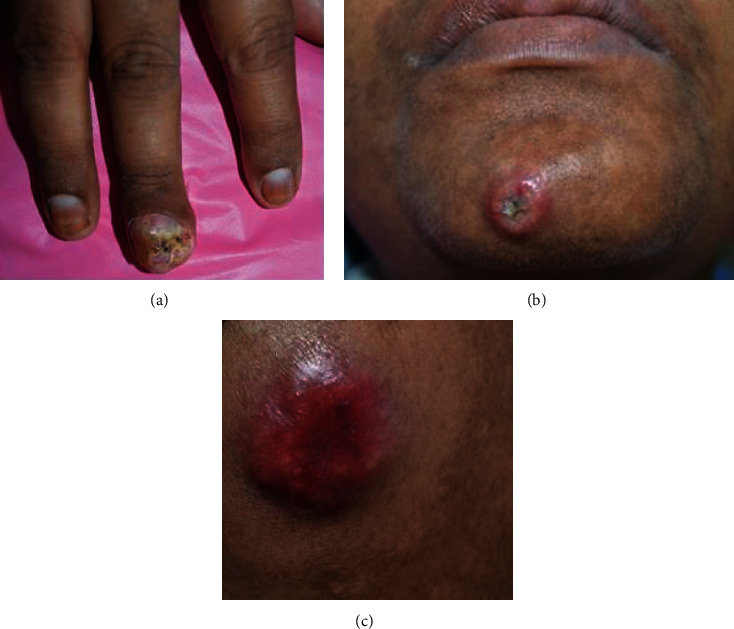
(a) Distal phalanx lesion on the third digit of the right hand. (b) and (c) Lesion with central necrotic crust involving the chin and forearm.

On intra-oral clinical examination, a dumbbell-shaped swelling of the gums was seen extending from the buccal aspect through the interdental space to the palatal aspect in the upper maxillary teeth (between 11 and 21). The swelling on the buccal aspect appeared smooth and shiny, while the palatal swelling had a more irregular surface. The swelling was reddish pink in color. The dimension of the buccal swelling was 16 mm × 11 mm and the palatal swelling was 13 × 12 mm approximately (Figures 1(a) and 1(b)). The patient reported that the swelling was initially small and grew at a rapid rate to the current size within a span of 2 weeks, causing an increase in space between the central incisors. The patient also had a history of occasional pain and bleeding from the palatal side of the swelling. Plaque and calculus deposits were present with generalized gingival inflammation. On general examination, the patient presented with cancer cell deposits, presenting as a painless nodular swelling with a central necrotic crust on the tip of the middle finger of the right hand involving the fingernail, chin, scalp, and the upper arm (Figure 2).

Radiographic analysis was done to check for bony involvement and periapical radiographs showed bone loss extending beyond the middle third of the root in relation to 11 and 21. Cone beam computed tomography (CBCT) evaluation revealed that the height of maximum bone loss in the mid-alveolus region of 11 and 21 measured about 10.80 mm (Figures 1(c) and 1(d)). A panoramic radiograph was also taken to rule out the presence of other lytic bony lesions involving the jaw bones. Prior to surgery, routine hematological investigations were done and the reports showed that the blood parameters were just above the normal range.

**Figure 3:**
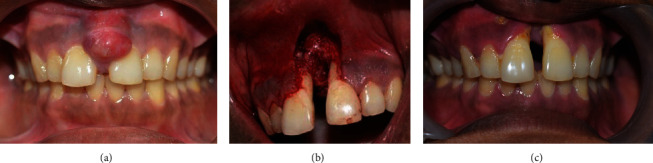
(a) Pre-operative view of growth. (b) Immediate post-operative view after excision of the lesion. (c) 3 months post-operative view.

A wide excision of the lesion was planned to remove the entire swelling with a 2 mm margin of healthy tissue using a 15c blade. On the removal of the lesion, the exposed underlying bone showed a moth-eaten appearance, which is characteristic of lytic lesions of the bone (Figure 3). The large defect was sealed with a gelatin sponge, sutures were given, and a periodontal dressing was placed to shield the surgical site. The patient was prescribed antibiotics and analgesics. Post-operative review after 3 months revealed satisfactory healing of the wound.

**Figure 4:**
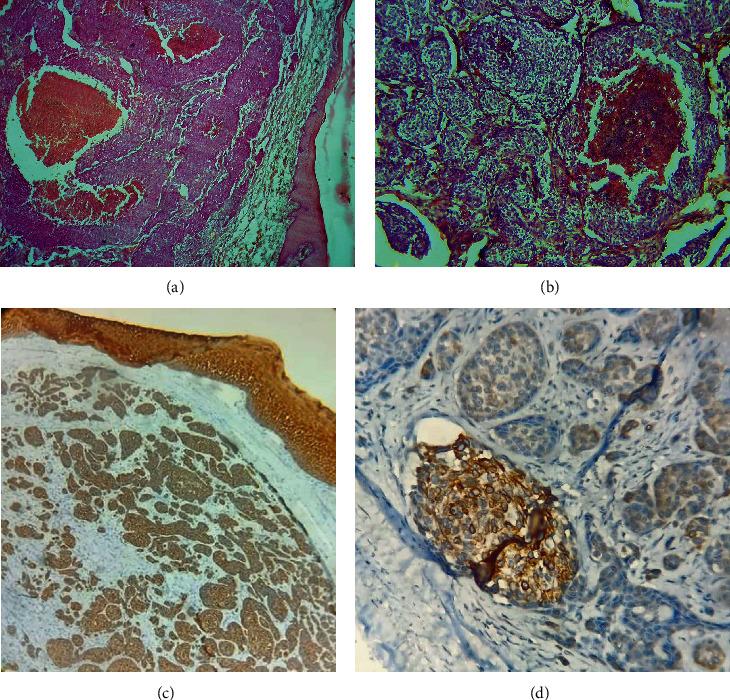
(a) Scanner view showing cohesive islands of tumour epithelial islands separated from the overlying surface epithelium by a grenz zone. Some tumour islands show central necrosis (4×); (b) Low power view shows cohesive islands of tumour epithelial islands and some tumour islands show central necrosis. (10×). (c) The surface epithelium and infiltrating epithelial tumour islands in the connective tissue were positive for Pan CK (4×). (d) IHC showing positive CK7 staining.

Histopathology of the hematoxylin and eosin-stained soft tissue section showed an atrophic ulcerated stratified squamous epithelium in association with a fibrovascular connective tissue. The connective tissue showed infiltrating solid islands of malignant epithelial tumour cells arranged in groups and clusters. The tumour cells were polygonal with eosinophilic cytoplasm and open-faced nuclei. Cells have pleomorphic hyperchromatic nuclei. The tumour cells showed numerous mitotic figures. Some of the dysplastic islands showed central necrosis resembling comedonecrosis. Focal areas of the tumour cells showed cytoplasmic clearing. The associated connective tissue was fibrovascular and scanty. The histopathological features were suggestive of infiltrating poorly differentiated adenocarcinoma (Figure 4). The tissue specimens were sent for immunohistochemistry (IHC) for confirmatory diagnosis. IHC evaluation showed that tumour cells were positive for cytokeratin (Pan CK) and specifically for CK7 while showing negative results for oestrogen receptor (ER), human epidermal growth factor receptor (HER)-2, GATA binding protein (GATA) 3, and thyroid transcription factor (TTF)1.

**Figure 5:**
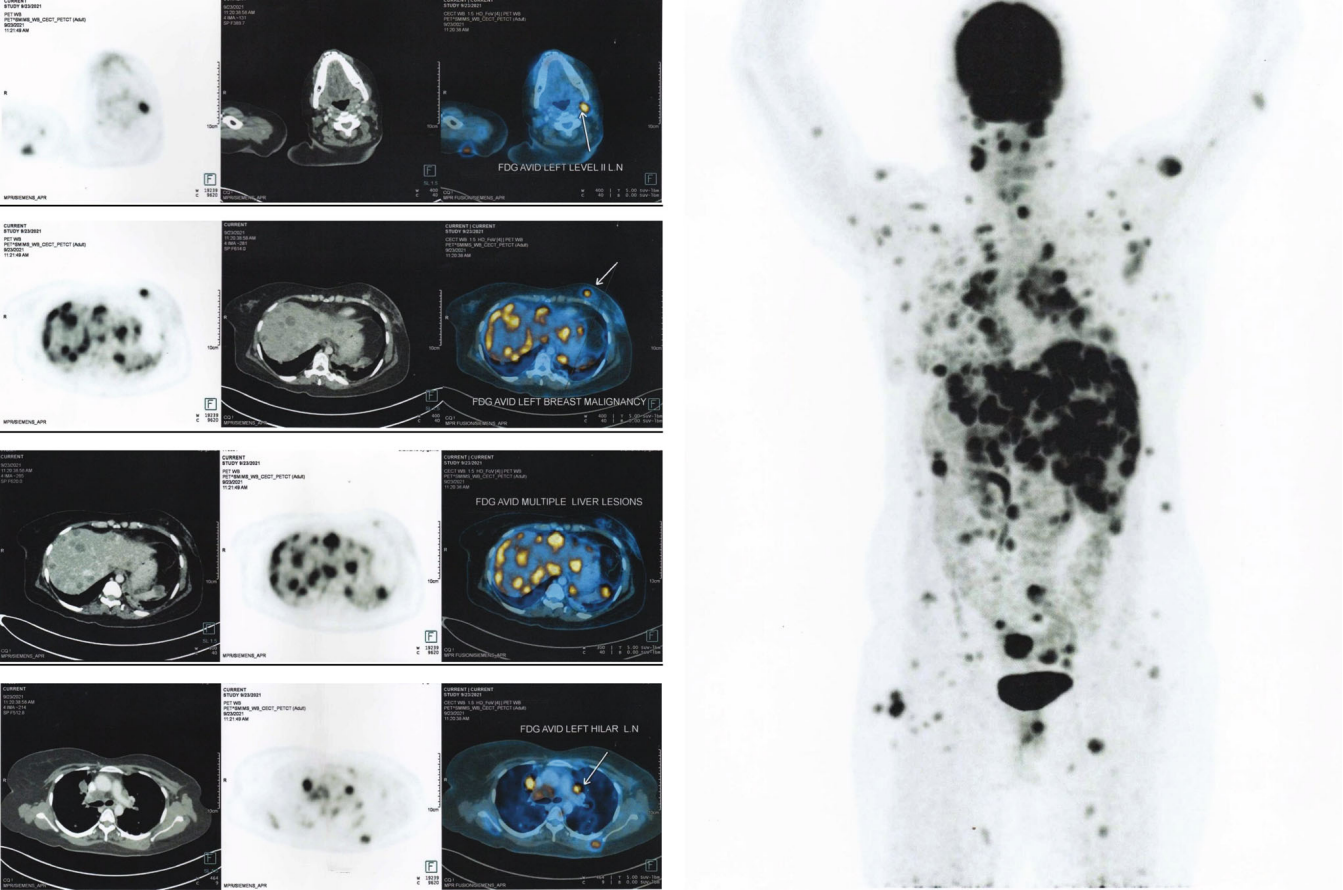
PET scan images showing extensive FDG avid metastases all over the body.

After excision of the gingival enlargement, a PET scan was done which revealed extensive Fluorodeoxyglucose (FDG) avid metastases in the brain, lung, liver, pancreas, nodes, subcutaneous and intramuscular soft tissue nodules, and sacrum (Figure 5). FDG avid lesions in both breasts were also seen. Compared with the previous PET-CT, the reports suggested progression of the disease. The patient was started on chemotherapy—Eribulin in view of disease progression. Unfortunately, fourteen months after the detection of the primary breast cancer tumour the patient passed away.

## 3. Discussion

Metastasis is said to represent the end stage of the invasion cascade, it includes the dissemination of tumour cells to distant organ sites and their adaptation to foreign tissue microenvironment [7]. Kwon et al. in 2006 described three criteria for the diagnosis of a metastatic tumour as (1) histological verification of the primary tumour, (2) the metastatic tumour and primary tumour must possess histological similarity, (3) exclusion of the possibility of direct spread from the primary tumour [10]. Hirshberg et al. in 2014 have described that a metastatic colony is due to a process known as the ‘invasion-metastasis cascade'. This involves a series of sequential steps including invasion through the surrounding extracellular matrix of the primary site, intravasation into the blood vessels, and the survival of circulating tumour cells (CTC) in circulation. The CTCs settle in the microvasculature of the target tissues and extravasate through the vessel wall. The cancer cells then reinitiate their proliferative properties at the target site leading to the formation of clinically detectable neoplastic lesions [11]. Metastasis to distant organs depends on the site and tissue-specific activities. It is now speculated that for a successful metastasis to occur, it needs a pre-metastatic niche, which allows the invading tumour cells to colonize, survive, and expand to form a macro-metastasis [12]. For men, the most common primary sites are the prostate, lung, liver, and kidney. For women, the most common primary sites are the breast, kidney, female genital organs, and colo-rectum [13]. Reports suggest that prostate and breast cancers usually metastasize to the jaw bones, while oral soft tissue metastases are frequently seen in lung carcinomas. Retrospective studies have reported a male predilection over females in metastases in the oral region [14]. Most of the patients presenting with oral cavity metastases are in the age group between 50 and 70 years, although variations in age have been reported [6]. However, in our case, the patient was only 37 years old.

Invasive ductal carcinoma is the most common form of breast cancer and accounts for 50–70% of invasive breast cancers [15]. Irani in 2016, in a review of 412 metastatic lesions that occurred in oral soft tissues, showed that gingiva was the most common site for metastases. The possible routes of oral metastasis include arterial, lymphatic, circulation, venous, and Batson's plexus. The majority of gingival metastases were reported in posterior areas. Anterior gingival metastases were seen only in 3.9% of cases [16]. In our case, the metastatic lesion was seen in the maxillary anterior region; a site that has not been commonly reported in the literature.

Allon et al. in 2014 have reported that in the soft tissues of the oral cavity, about 60% of the metastatic lesions are found in the attached gingiva followed by the tongue (18%). They also reported that in the presence of teeth, 80% of patients had metastasis in the gingiva when compared with one-third of edentulous patients [7]. The presence of inflammation in conditions such as gingivitis and periodontitis is speculated to be the critical factor regulating metastasis. Inflammation in the gingiva leads to vascular network proliferation as well as destruction of the basement membrane, which could attract metastatic cells [17]. There is growing evidence of the role of gingival inflammation acting as a cofactor in attracting metastatic tumour cells and resulting in gingival metastatic lesions. Spano and Zollo in 2012 have suggested a metastatic niche model, which suggests that it is necessary for the presence of a suitably conducive microenvironment referred to as the pre-metastatic niche for tumour cells to invade and proliferate at secondary sites [18]. There has been speculation on the role of gingival inflammation in the dissemination of gingival metastasis. Chronically inflamed gingiva may provide a suitable niche for metastatic tumour cells to colonize and proliferate [7]. The inflamed gingiva provides a permissive niche for metastatic tumour cells, which favours angiogenesis, immune evasion, and formation of a supporting stroma [19]. Periodontal disease results in the release of proinflammatory cytokines such as interleukin 1 (IL-1) and tumour necrosis factor *α* (TNF-*α*) which stimulate angiogenesis and formation of extracellular matrix, which facilitates metastatic progression. The cytokines may also induce or attract tumour-associated macrophages [20].

The involvement of the gingiva and underlying bone in metastatic lesions is an area of controversy. It remains unclear whether cancer cells initially metastasize to the gingiva or whether it is a bone metastasis, which subsequently involves the gingiva or whether the patient develops both metastases simultaneously [21]. In our case, the clinical presentation was that of a gingival swelling which on surgical excision revealed underlying bone destruction.

It is clinically difficult to distinguish between gingival metastatic lesions and benign lesions. Some of the benign inflammatory lesions include gingival hyperplasia, fibrous epulis, and pyogenic granuloma. However, malignancy needs to be suspected if there is a definitive diagnosis of a primary tumour elsewhere in the body. Other clinical presentations for suspecting a malignant lesion could be a rapid rate of gingival overgrowth, abnormal bleeding tendency, and unexplained rapid changes in occlusion. Gingival metastatic lesions may cause progressive pain, discomfort, bleeding, superinfection, interference with mastication, dysphagia, and disfigurement [13]. It is therefore mandatory to perform a biopsy of any suspicious overgrowth. The histopathological features in our case were suggestive of infiltrating poorly differentiated adenocarcinoma.

To recognize a lesion as a metastasis, it is important to differentiate between primary intra-oral malignancies from a metastatic tumour. Soft tissue malignant tumours rarely originate intra-orally and therefore should be worked up as a metastatic lesion rather than a primary tumour. Immunohistochemical (IHC) phenotyping of metastatic tumour cells is now considered helpful in determining the site of the primary tumour [22]. If the patient presents with a history of cancer, it is important that the histopathological features of the metastatic lesion should be compared with those of the primary tumour. Special staining techniques like IHC can be helpful in identifying the nature of the lesion. Lesimple et al. in 2003 have given clinical practical guidelines for the diagnosis of carcinomas of an unknown primary site in which they mentioned an initial IHC panel, which includes antibodies against epithelial antigens (broad spectrum anti-pan-cytokeratin antibody cocktail), lymphoid antigen (CD 45/CD20/CD3) and melanocyte-differentiation antigens (S100, SOX 10) [23]. IHC is used to distinguish three histopathological subtypes among carcinoma metastases: (1) adenocarcinoma (in about 50% of cases), (2) poorly differentiated carcinoma (in about 30% of cases), and (3) squamous cell carcinoma (in about 15% of cases). Based on the expression profiles of CK7 and CK20, different subsets of carcinomas have been defined [24]. Studies have shown that the different expression patterns of CK (7 and 20) are among the most discriminant markers in the differential diagnosis of breast adenocarcinoma. The additional markers include ER/PR, GCDFP-15, and mammaglobin [25]. A detailed mapping of cytokeratin 20 (CK20) and cytokeratin 7 (CK7) profiles will help in directing the clinical investigations in most of the common types of adenocarcinomas [26]. The majority of oral metastatic tumours are of epithelial origin with adenocarcinoma being the most common type [4]. Among the breast cancers, adenocarcinoma is said to be one of the most common sources of distant metastases.

Several studies have shown that the CK20^−^/CK7^+^ immunophenotype are typical for breast carcinomas. Malzahn et al. in 1998 have evaluated the cytokine profiles in a series of 101 Ductal breast carcinomas. They have reported only three CK20^+^ cases, all of which were poorly differentiated, whereas the majority of the tumours were CK7^+^ [27]. Tibor Tot in 1999, in a study of 123 invasive breast carcinomas, 92% metastases showed to be CK 20 negative while 98% of the tumours were CK7 positive [28]. The diagnostic workflow suggests that once the CK7/CK20 expression profile is detected, complementary organ-specific antibody studies will allow precise guidance towards the primary origin of the tumour. Mhawech-Fauceglia et al. in 2012 have mentioned that the most classical IHC markers that can be used to identify the adenocarcinoma of unknown origin along with CK7^+^/CK20^−^ profile are thyroid transcription factor (TTF) 1, GATA binding protein (GATA) 3, paired box gene (PAX) 8, and Wilms Tumour (WT) 1 [29].

If the primary site of origin of tumour is the breast and with expression of CK7^+^/CK20^−^ profiles, IHC tumour staining patterns include oestrogen receptor (ER)+/Progesterone receptor (PgR)^+^, GATA binding protein (GATA) 3^+^, gross cystic disease fluid protein (GCDFP) 15^−/+^, mammaglobin (MGB)^+/−^, thyroid transcription factor (TFF) 1^–^ [30]. In our case, IHC evaluation showed that tumour cells are positive for CK7 and negative for CK20. The cells were also negative for ER, Her-2, GATA 3 and TTF1. Janick Selves has reported in his article that around 5% of metastatic tumours especially poorly differentiated ones will not provide the clues to its origin during IHC investigations. They have also reported that some tumour-specific antibodies may show a less intense signal or no signal at metastatic sites [31]. In our case we had an identifiable primary site with metastatic deposits showing histologic features resembling ductal carcinoma and correlating with the IHC findings, a diagnosis of metastasizing poorly differentiated adenocarcinoma of the breast was made.

Metastatic tumours in the oral cavity do not usually present typical pathognomonic radiographic appearances. Most often, osteolytic changes with ill-defined and irregular margins may be seen. In the present case, there was a solitary gingival enlargement in the maxillary anterior region, radiographic evidence of bone loss was seen, which was clinically confirmed after excision of the lesion. In the majority of the oral metastatic tumours reported in the literature, there is a known primary tumour, which has been diagnosed. In our patient, breast cancer was the primary tumour and multiple metastatic lesions were present throughout the body. However, in some patients the oral lesions may be the first presentation with the primary tumour being unknown. This presents a clinical challenge. The prognosis and treatment are said to depend on the site of the primary tumour and the degree of spread of metastasis [32]. The time between the diagnosis of oral metastatic lesions and the patient's death varies from month to years, with a median survival ranging between a few months to 5 years. A mean survival of 6 to 9 months has been reported in studies by Hirshberg et al., van der Waals et al., and Oliver-Puigdomènech et al. [13, 33, 34].

In the present case, a 37-year-old patient presented with invasive ductal carcinoma of the breast as the primary tumour. Within a period of 9 months, PET scan revealed extensive metastases in the brain, lung, liver, pancreas, nodes, subcutaneous and intramuscular soft tissue nodules, and sacrum. She presented with a solitary gingival enlargement in the maxillary anterior attached gingiva region. The time interval from the detection of primary tumour and detection of oral metastasis was 9 months. The lesion was excised and was histopathologically diagnosed as infiltrating poorly differentiated adenocarcinoma. She was on chemotherapy, but the extensive spread of cancer all over the body and as oral metastasis is usually seen late in disease progression meant that her disease prognosis was poor. The time interval between the diagnosis of the primary tumour and death in our case was 14 months.

## 4. Conclusion

This paper presents the clinicopathological features of gingival metastasis from breast cancer in a 37-year-old woman. The rarity of this case was that it occurred in a young patient involving the maxillary anterior gingiva, which has not been commonly reported in the literature. Early diagnosis and treatment of oral cavity metastases presents a therapeutic challenge for the clinician, which can play an important role in improving the patient's quality of life and prolonging survival. It is mandatory to differentiate malignancies from inflammatory and reactive lesions of the gingiva. Therefore, it is of utmost importance to take a detailed patient history, a thorough clinical and radiographic examination in case of gingival swelling. We therefore recommend a biopsy for any gingival swelling in the context of previous malignancy and an IHC evaluation to determine the site of primary tumour.

## Abbreviations

IHC: Immunohistochemistry
PET: Positron emission tomography
cGY: Centigray
CBCT: Cone beam computed tomography
CK: Cytokeratin
ER: Oestrogen receptor
HER-2: Human epidermal growth factor receptor 2
GATA 3: GATA binding protein 3
TTF 1: Thyroid transcription factor 1
FDG: Fluorodeoxyglucose
CTC: Circulating tumour cells
IL-1: Interleukin 1
TNF-*α*: Tumour necrosis factor *α*
CD 45/CD20/CD3: Cluster of Differentiation 45/20/3
S100 protein: These proteins are soluble in 100%, i.e. saturated, ammonium sulfate at neutral pH
SOX 10: Sry-related HMg-Box gene 10.
